# Anticancer effects of licochalcones: A review of the mechanisms

**DOI:** 10.3389/fphar.2023.1074506

**Published:** 2023-01-23

**Authors:** Nan Deng, Mingming Qiao, Ying Li, Fengyan Liang, Jingjing Li, Yanfeng Liu

**Affiliations:** ^1^ Dongzhimen Hospital, Beijing University of Chinese Medicine, Beijing, China; ^2^ Chongqing Institute for Food and Drug Control, Chongqing, China

**Keywords:** anti-cancer, mechanism, licochalcone, licochalcone A, autophagy, apoptosis, cell cycle

## Abstract

Cancer is a disease with a high fatality rate representing a serious threat to human health. Researchers have tried to identify effective anticancer drugs. Licorice is a widely used traditional Chinese medicine with various pharmacological properties, and licorice-derived flavonoids include licochalcones like licochalcone A, licochalcone B, licochalcone C, licochalcone D, licochalcone E, and licochalcone H. By regulating the expression in multiple signaling pathways such as the EGFR/ERK, PI3K/Akt/mTOR, p38/JNK, JAK2/STAT3, MEK/ERK, Wnt/β-catenin, and MKK4/JNK pathways, and their downstream proteins, licochalcones can activate the mitochondrial apoptosis pathway and death receptor pathway, promote autophagy-related protein expression, inhibit the expression of cell cycle proteins and angiogenesis factors, regulate autophagy and apoptosis, and inhibit the proliferation, migration, and invasion of cancer cells. Among the licochalcones, the largest number of studies examined licochalcone A, far more than other licochalcones. Licochalcone A not only has prominent anticancer effects but also can be used to inhibit the efflux of antineoplastic drugs from cancer cells. Moreover, derivatives of licochalcone A exhibit strong antitumor effects. Currently, most results of the anticancer effects of licochalcones are derived from cell experiments. Thus, more clinical studies are needed to confirm the antineoplastic effects of licochalcones.

## 1 Introduction

Cancer seriously affects human health, and in the 21st century, it is expected to become the main cause of death in every country/region (Bray et al., 2018; Keshavarz-Fathi and Rezaei, 2021). Licorice is a widely used traditional Chinese medicine with a variety of pharmacological properties including anti-inflammatory, antioxidant, antidiabetic, anti-asthmatic, and anticancer activities. The pharmacological effects are related to the flavonoids it contains, among which licochalcones have considerable antitumor activity (Hosseinzadeh and Nassiri-Asl, 2015; Pia et al., 2019).

**FIGURE 1:**
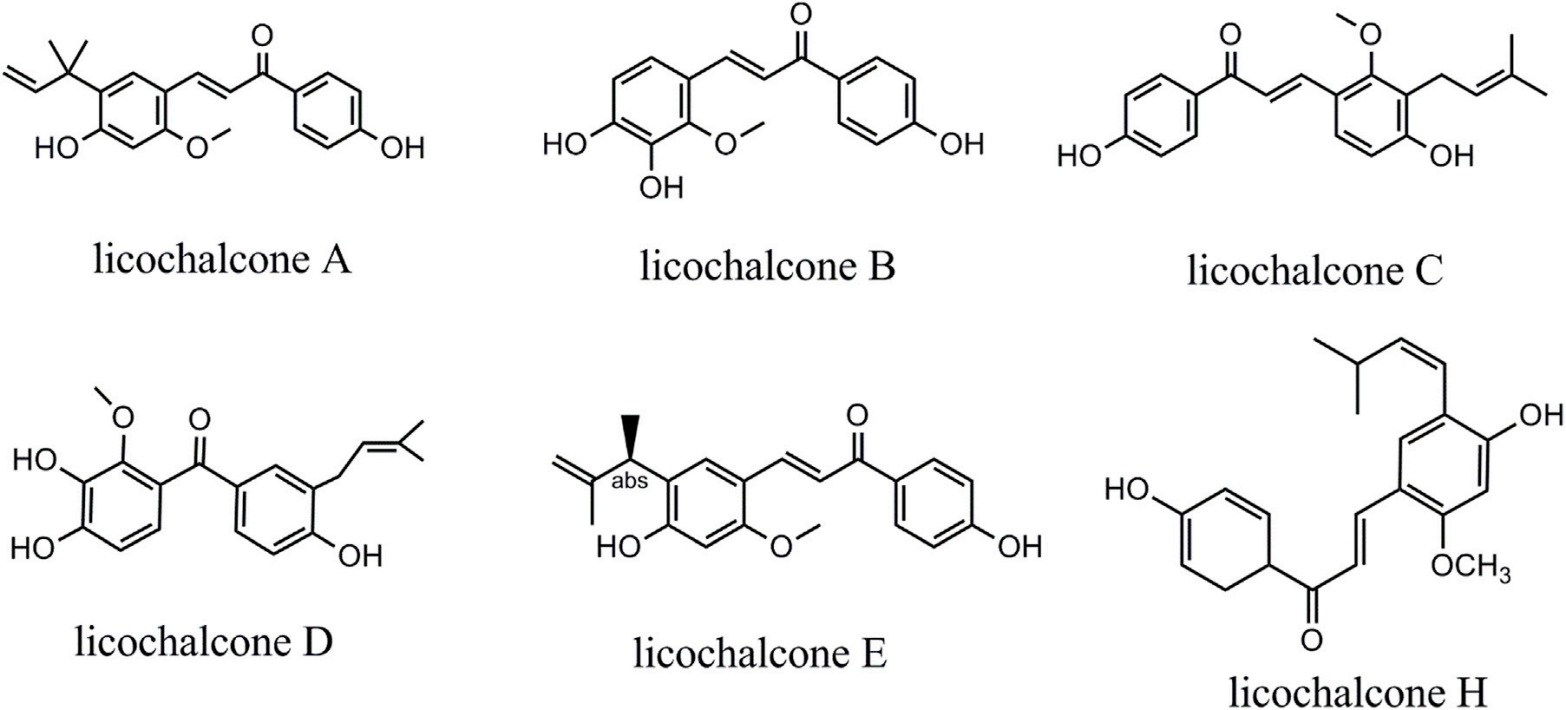
Chemical structure of licochalcones.

This review systematically reviewed the evidence on the anticancer effects of licochalcones. Licochalcones in licorice comprise licochalcone A (LA), licochalcone B (LB), licochalcone C (LC), licochalcone D (LD), licochalcone E (LE), and licochalcone H (LH) (Figure 1). These licochalcones can activate the mitochondrial apoptosis pathway and the death receptor pathway, promote autophagy-related protein expression, inhibit cell cycle protein expression, and regulate cancer migration-related protein expression *via* multiple signaling pathways, including EGFR/ERK, PI3K/Akt/mTOR, p38/JNK, JAK2/STAT3, MEK/ERK, Wnt/β-catenin, and MKK4/JNK signaling pathways. Via these mechanisms, licochalcones can induce autophagy and apoptosis in cancer cells, as well as inhibit cancer cell proliferation, migration, and invasion. In tumor tissues, LA can also inhibit angiogenesis and the cellular efflux of anticancer drugs. At present, the antineoplastic effects of LA are most prominent. Various LA derivatives exhibit stronger antitumor effects than LA, and exploring the anticancer mechanisms of these licochalcone derivatives may be a focus of future studies. As most of the licochalcone-induced effects have been examined in cell experiments, more clinical studies are needed to confirm the antitumor effects of licochalcones in the future. Thus, this paper systematically reviewed the literature regarding licochalcone-induced anticancer effects with a focus on LA to provide an overview of the current knowledge and guide further basic and clinical research.

## 2 Anticancer effects of licochalcones on tumor cells

### 2.1 Lung cancer

Epidemiological studies have shown that approximately 18 million people are diagnosed with lung cancer each year, and 16 million of them die from this disease (Chen et al., 2014; Hirsch et al., 2017). Licochalcones can induce apoptosis and autophagy, and they inhibit the proliferation, migration, and invasion of lung cancer cells (Keshavarz-Fathi and Rezaei, 2021).

#### 2.1.1 Induction of autophagy

LC3-II is considered to be a characteristic protein of the autophagy process. LA (10–15 μM) can induce autophagy in A549 and H1299 lung cancer cells by increasing the LC3-II/LC3-I ratio, as well as the levels of the autophagy-related proteins ATG5, ATG7, and P62. LA-induced increases in CHOP expression also promote autophagy (Tang et al., 2016; Lin et al., 2019a). Furthermore, LA-induced autophagy in lung cancer cells is associated with the induction of endoplasmic reticulum stress. LA (10 μM) enhances the expression of miR-144-3p, causes unfolded protein response, and triggers autophagy by promoting the accumulation and expression of ATG1, ATG3, ATG6, and ATG16 *via* activation of the PERK/ATF4/CHOP signaling pathway (Chen et al., 2018).

#### 2.1.2 Induction of apoptosis

Activation of the mitochondrial apoptosis pathway is closely related to mitochondrial dysfunction. LA (2.5–25 μM) inhibited ATP production and caused mitochondrial dysfunction in H1299 and H322 lung cancer cells by inhibiting hypoxia-induced HIF-1α accumulation and the expression of target genes *GLUT1* and *PDK1*, which induced activation of the mitochondrial apoptosis pathway and apoptosis of cancer cells (Park et al., 2021). LA (10–15 μM) can activate the mitochondrial apoptosis pathway and induce apoptosis in H460 and A549 lung cancer cells by decreasing the levels of Bcl-xL and Bcl-2 while increasing the levels of Bad, Bax, cleaved PARP, and caspase-3 (Tang et al., 2016; Qiu et al., 2017; Lin et al., 2019a). In addition, the survivin protein inhibits caspase-3 activity and prevents cancer cell apoptosis. LA (5–50 μM) downregulated the expression of survivin by inhibiting the EGFR signaling pathway and its downstream kinases ERK1/2 and AKT in H3255, HCC827, H1975, and A549 lung cancer cells (Gao et al., 2021). LB (5–15 μM) inhibited the EGFR and MET signaling pathways and induced mitochondrial dysfunction and endoplasmic reticulum stress in HCC827 lung cancer cells, which induced the loss of MMP, release of cytochrome c, and increased expression of caspases (Oh et al., 2019a).

#### 2.1.3 Cell cycle block

By inhibiting the expression of MDM2, cyclin B1, CDC2, and CDC25C, LA (10–15 μM) led to cell cycle arrest of H460 and A549 lung cancer cells at the G2/M phase (Qiu et al., 2017; Lin et al., 2019a). Heng et al. found that decreases in the proliferation of lung cancer cells by LA were related to the inhibition of the Wnt/β-catenin signaling pathway (Heng and Cheah, 2021). LB (5–15 μM) caused cell cycle arrest of HCC827 lung cancer cells at the G2/M phase by decreasing the expression of cyclinB1 and CDC2 proteins while increasing p27 expression (Oh et al., 2019a).

#### 2.1.4 Inhibition of migration and invasion

LA (2–20 μM) inhibited the AKT signaling pathway and the expression of the downstream transcription factor Sp1, which reduced the levels of MMP-1 and MMP-3 and inhibited the migration and invasion of A549 and H460 lung cancer cells (Huang et al., 2014).

#### 2.1.5 Activation of the immune system

PD-L1 is a key immune checkpoint, and reducing the production of PD-L1 can play a role in immunotherapy. LA (10–50 μM) inhibited the expression of PD-L1 and thereby induced the production of reactive oxygen species (ROS) in A549 lung cancer cells, which inhibited the phosphorylation of 4EBP1, activated the PERK/eIF2α pathway, and ultimately induced apoptosis in cancer cells (Yuan et al., 2021).

### 2.2 Liver cancer

Licochalcones can induce apoptosis and inhibit the proliferation, migration, and invasion of hepatocellular carcinoma cells (Keshavarz-Fathi and Rezaei, 2021).

#### 2.2.1 Induction of apoptosis

By inhibiting the PI3K/Akt/mTOR signaling pathway, LA (5–20 μM) activated the mitochondrial apoptosis pathway and promoted the expression of Bax, Bad, and caspase-3, thereby inducing apoptosis in HepG2 cells (Wu et al., 2019). In addition, LA (1–50 μM) induced endoplasmic reticulum stress in HepG2 cells by inducing phosphorylation of VEGFR2, c-Met receptor, and PLCγ1 and enhancing the cytosolic Ca^2+^ release from the endoplasmic reticulum, which subsequently induced ROS accumulation, the expression of CHOP, as well as caspase-4, -9, and -3, and ultimately cell apoptosis (Choi et al., 2014). Wang et al. found that the production and accumulation of intracellular ROS also activate the mitochondrial apoptosis pathway and increased the expression of Bad, Bax, Bak, PUMA, and caspase-3. The production of intracellular ROS in HepG2 cells is involved in the LA-induced (5–50 μM) downregulation of PDK1 and rubicon by activating the ULK1/Atg13 signaling pathway and increasing the expression of TSC1/2, PRAS40, CTMP, and PP2A. Moreover, LA activates the death receptor pathway and caspase cascade by increasing the expression of DR3, DR5, and Fas. LA also decreases the expression of the survival factor PKCε, p70S6K, and Akt. Through these effects, LA (30–70 μM) finally induces apoptosis in HepG2 cells (Niu et al., 2018; Wang et al., 2018). LA (70 μM) can induce apoptosis of HepG2 cells by regulating the MAPK and FoxO signaling pathways (Wang et al., 2021). Likewise, LB (10–120 μM) induces apoptosis in HepG2 cells by activating the extrinsic apoptotic pathway (i.e., increasing the expression of TNFR1, Fas, Fas-L, caspase-8, JUN, and Fos) and mitochondrial apoptosis pathway (i.e., increasing the expression of Bak, caspase-9, and caspase-3) (Wang et al., 2019). LB (120 μM) can promote the apoptosis of HepG2 cells by regulating microRNAs (miRNAs) including miR-29b-3p and miR-96-5p (Wang and Wang, 2021). By inhibiting the expression of EGFR and MET, promoting the accumulation of intracellular ROS, activating the mitochondrial apoptosis pathway, increasing the expression of Bid, Bad, and cleaved PARP, and reducing the expression of Bcl-xL and Mcl-1, LD (5–20 μM) induced apoptosis in HCC827 cells (Oh et al., 2020).

#### 2.2.2 Cell cycle block

LA (30–70 μM) blocked the cell cycle of HepG2 cells at the G2/M transition by increasing the expression of Weel, P21, and JNK1 while decreasing the expression of survivin, cyclin B1, cyclin D1, and CDK1. Further research showed the observed effects were related to the inhibition of the p38/JNK/ERK signaling pathway (Chen et al., 2017; Wang et al., 2018). By decreasing the expression of CDK1, cyclin B1, CHK2, CDC14B, and CDC7 and increasing the expression of p21, LB (10–120 μM) caused the cell cycle arrest of HepG2 cells at the G2/M phase (Wang et al., 2019). LB (10–20 μM) blocked the cell cycle of HepG2 and Huh7 cells at the G2/M phase *via* increased p27 expression and decreased cyclin B1 and Cdc2 levels (Zhang et al., 2022). By decreasing the expression levels of cyclin B1 and CDC2 and increasing those of p21 and p27, LD (5–20 μM) induced a cell cycle arrest of HCC827 cells at the G2/M phase (Oh et al., 2020).

#### 2.2.3 Inhibition of migration and invasion

By downregulating the expression of uPA and MMP9 through inhibition of the MKK4/JNK and NF-κB signaling pathways, LA (5–20 μM) inhibited the migration and invasion of HA22T/VGH and SK-Hep-1 cells (Tsai et al., 2014; Wu et al., 2018a).

### 2.3 Breast cancer

Research has shown that licochalcones can induce apoptosis and autophagy and inhibit the proliferation, migration, invasion, and angiogenesis of breast cancer cells (Keshavarz-Fathi and Rezaei, 2021).

#### 2.3.1 Induction of autophagy

LA (5–50 μM) can inhibit the PI3K/Akt/mTOR signaling pathway, which increases the expression of LC3-II protein and ultimately induces autophagy in MCF-7 cells (Xue et al., 2018).

#### 2.3.2 Induction of apoptosis

By inhibiting the PI3K/Akt/mTOR signaling pathway, LA (5–50 μM) activated the mitochondrial apoptosis pathway, reduced the expression of Bcl-2, and promoted the expression of Bax and caspase-3, thereby inducing the apoptosis in MCF-7 cells (Huang et al., 2019). By decreasing the mitochondrial membrane potential and inducing ROS production, LA (5–50 μM) upregulated the expression of Bid, Bad and cleaved PARP, downregulated the expression of Bcl-2 and Bcl-xL, and induced the apoptosis in MCF-7 and MDA-MB-231 cells. The authors suggested that these effects were related to the inhibition of Sp1 (Kang et al., 2017a). Via increases in the level of acylcarnitine and inhibition of prostaglandin reductase 1 expression, LA (1–100 μM) caused respiratory dysfunction in HCC38 TNBC cells and thus induced cancer cells apoptosis (Roberts et al., 2017). LB (10–50 μM) induced apoptosis in MCF-7 cells by increasing the expression of Bid, Bad, cleaved PARP, and caspase-3 while decreasing the expression of Bcl-2 (Yu et al., 2016).

#### 2.3.3 Cell cycle block

By downregulating the expression of Sp1, LA inhibited the proliferation of MCF-7 and MDA-MB-231 cells *via* increases in the expression of the proteins p21 and p27 and decreases in the expression of Mcl-1 and survivin (Kang et al., 2017a). LA (5–15 μg/mL) also blocked the cell cycle of MCF-7 cells at the G1 phase *via* reduced cyclin D1 expression (Bortolotto et al., 2017). By reducing the expression of cyclin D1 and increasing the expression of p21, LA (5–50 μM) led to cell cycle arrest of MCF-7 cells at the G0/G1 phase (Huang et al., 2019). The overexpression of PRMT6 in human breast cancer is related to tumorigenesis (Cheng and Bedford, 2011), and LA (10–100 μM) caused the cell cycle arrest of MCF-7 cells at the G2/M phase by inhibiting PRMT6 expression and subsequently p53 expression (Gong et al., 2020). By downregulating the expression levels of cyclin A, CDK2, and CDC25A and increasing the level of p21, LB (10–50 μM) induced the cell cycle arrest of MCF-7 cells at the S phase (Yu et al., 2016). By decreasing the expression of CDK4, CDK2, cyclin A, and cyclin D1, LE (7–14 mg/kg) inhibited the proliferation of MDA-MB 231 cells in mice (Kwon et al., 2013).

#### 2.3.4 Inhibition of migration and invasion

LA (5–40 μΜ) inhibited the migration and invasion of MDA-MB-231 cells by suppressing the expression of E-cadherin and vimentin *via* inhibition of the p38 MAPK and AKT signaling pathways (Huang et al., 2019). By decreasing the expression of uPA and MMP-9, LE (7–14 mg/kg) inhibited the migration and invasion of MDA-MB 231 cells in mice (Kwon et al., 2013).

#### 2.3.5 Inhibition of angiogenesis

Via downregulation of VEGF-A, LE (7–14 mg/kg) inhibited angiogenesis in cancer tissue in a xenograft mouse model using MDA-MB 231 breast cancer cells (Kwon et al., 2013).

### 2.4 Oral carcinoma

Head and neck cancer is the seventh most common cancer in the world, and almost half of the tumors are oral carcinomas. Oral squamous cell carcinoma is the most common form of oral cancer, with a poor prognosis and high mortality (Vitorio et al., 2020). Licochalcones can induce apoptosis and inhibit the proliferation, migration, and invasion of oral carcinoma cells (Keshavarz-Fathi and Rezaei, 2021).

#### 2.4.1 Induction of apoptosis

By activating the mitochondrial apoptosis pathway and increasing the expression of Bax, Bid, cleaved PARP, and caspase-3, LA (10–40 μM) induced apoptosis in HN22 and HSC4 cells (Cho et al., 2014). Similarly, by activating the mitochondrial apoptosis pathway and the caspase cascade, LA (5–100 μg/mL) induced apoptosis in SCC-25 cells (Zeng et al., 2014). In addition to its effects on the mitochondrial apoptosis pathway, LA (IC_50_ = 50 μM) activated the FasL-mediated death receptor pathway and upregulated the caspase-8 and -3 levels in KB cells (Kim et al., 2014). In pharyngeal squamous carcinoma FaDu cells, LA (10–100 μM) activated the death receptor pathway by increasing the expression of TRAIL *via* stimulation of the ERK1/2 and p38 MAPK signaling pathways. Moreover, the activation of the ERK1/2 and p38 MAPK signaling pathways upregulated the p53 expression, thereby increasing the levels of Bad, Bax, caspase-3, -8, and -9, as well as the release of cytochrome c (Yang et al., 2014; Park et al., 2015). LB (10–30 μM) can also activate the death receptor pathway (i.e., increased expression of DR 4 and DR5) and induce endoplasmic reticulum stress (i.e., increased CHOP expression) in HN22 and HSC4 cells, which causes mitochondrial membrane depolarization, upregulates the expression of Bax, cleaved PARP, and caspase-3, and downregulates the expression of survivin, Bcl-xL, and Mcl-1 (Oh et al., 2016). By inhibiting the JAK2/STAT3 signaling pathway leading to activation of the death receptor pathway (i.e., increased expression of DR4 and DR5), endoplasmic reticulum stress (i.e., elevated ROS and CHOP levels), and mitochondrial apoptosis pathway (i.e., upregulated expression of p21, Bax, Bid, Apaf-1, and cleaved PARP; downregulated expression of Bcl-2, Mcl-1, and survivin), LC (10–50 μM) and LD (10–30 μM) induced apoptosis in HN22 and HSC4 cells (Oh et al., 2018; Seo et al., 2019). LD (12.5–50 μg/mL) induced apoptosis in pharyngeal squamous carcinoma FaDu cells by increasing Fas-L, p53, Bax, Bid, and caspase-3 expression while decreasing the expression of Bcl-2 by activating the death receptor and mitochondrial apoptosis pathways (Yu et al., 2017). LH (10–30 μM) induced apoptosis in HSC2 and HSC3 cells by downregulating the expression of Bcl-2 and Bcl-xL and upregulating the expression of Bax and Bad *via* inhibition of Matr3 expression (Nho et al., 2019). LH (5–20 μM) can also induce apoptosis in HN22 and HSC4 cells by activating the death receptor pathway, endoplasmic reticulum stress, and mitochondrial apoptosis pathway *via* inhibition of the JAK/STAT3 signaling pathway (Oh et al., 2019b).

#### 2.4.2 Cell cycle block

LA (5–100 μg/mL) led to cell cycle arrest of SCC-25 cells at the S and G2/M phases (Zeng et al., 2014). Further research showed that LA (10–40 μM) inhibited the proliferation of HN22 and HSC4 cells by inhibiting Sp1 expression and regulating its downstream proteins including p27, p21, and cyclin D1 (Cho et al., 2014). Both LB and LD (10–30 μM) can induce G1 phase arrest in HN22 and HSC4 cells by increasing the p21 and p27 levels and decreasing the level of cyclin D1 (Oh et al., 2016; Seo et al., 2019). Likewise, LH (5–20 μM) induced G1 phase arrest in HN22 and HSC4 cells by increasing the p21 and p27 levels and decreasing the level of cyclin D1 *via* inhibition of the JAK/STAT3 signaling pathway (Oh et al., 2019b).

#### 2.4.3 Inhibition of migration and invasion

LA (25–100 μM) inhibited the migration and invasion of SCC4 and CAL-27 cells by downregulating the IGF-1, MMP-2, and MMP-9 levels *via* inhibition of the PI3K/AKT signaling pathway (Hao et al., 2019). LA (25–100 μg/mL) can also inhibit the migration and invasion of SCC-25 cells by decreasing MMP-2 expression while increasing the levels of TIMP and E-cadherin (Shen et al., 2014).

### 2.5 Esophageal cancer

Esophageal cancer belongs to the head and neck cancers and is the sixth leading cause of cancer-related death. Its incidence rate increases every year (Zhang et al., 2021). Licochalcones can induce apoptosis and inhibit proliferation in esophageal cancer cells (Keshavarz-Fathi and Rezaei, 2021).

#### 2.5.1 Induction of apoptosis

LB (5–20 μM) induced apoptosis in KYSE-450 and KYSE-510 cells by inhibiting the JAK2/STAT3 signaling pathway and decreasing the expression of Mcl-1 (Song et al., 2020). Via inhibition of the JNK/p38 MAPK signaling pathway, LC (10–50 μM) induced endoplasmic reticulum stress (i.e., increased GRP78 and CHOP expression) and activated the mitochondrial apoptosis pathway in various esophageal squamous carcinoma cells, including KYSE-30, KYSE-70, KYSE-410, KYSE-450, and KYSE-510 cells (Kwak et al., 2020a). LH (5–20 μM) induced the apoptosis in KYSE-30 and KYSE-450 cells by activating the death receptor pathway, mitochondrial apoptosis pathway, and endoplasmic reticulum stress *via* increased DR4, DR5, CHOP, p21, Bax, Bid, Apaf-1, and cleaved PARP expression, stimulated ROS production, and decreased expression of Bcl-2, Mcl-1, and survivin (Kwak et al., 2020b).

#### 2.5.2 Cell cycle block

LB (5–20 μM) induced G2/M phase cell cycle arrest in KYSE-450 and KYSE-510 cells (Song et al., 2020). LH (5–20 μM) blocked the cell cycle of KYSE-30 and KYSE-450 cells at the G2/M phase by downregulating CDC2 and cyclin B1 expression and upregulating p21 and p27 (Kwak et al., 2020b).

### 2.6 Gastric cancer

Stomach cancer is the fifth most commonly diagnosed cancer and the third leading cause of cancer death (Bray et al., 2018). Research has shown that licochalcones can induce apoptosis and inhibit proliferation in gastric cancer cells (Keshavarz-Fathi and Rezaei, 2021).

#### 2.6.1 Induction of apoptosis

By activating the ERK, JNK, and p38 MAPK signaling pathways and inhibiting the PI3K/AKT signaling pathway, LA (20–100 μM) promoted the intracellular ROS generation and stimulated both the mitochondrial apoptosis pathway and caspase cascade in BGC-823 cells (Hao et al., 2015). Via activation of the mitochondrial apoptosis pathway, induction of Bax, Bad, cleaved PARP, caspase-3, -8, and -9 expression, and decrease in Bcl-2 expression, LA induced the apoptosis in five gastric cell lines including GES-1, MKN-28, SGC7901, AGS, and MKN-45 cells. The respective IC_50_ values of LA were 92.7, 42.0, 40.8, 41.1, and 40.7 μM (Xiao et al., 2011; Lin et al., 2017). Moreover, the inhibitory effect of LA (10–50 μM) on the AKT signaling pathway can downregulate the expression of hexokinase 2A and inhibit glycolysis, thereby inducing apoptosis of MKN45 and SGC7901 cells (Wu et al., 2018b).

#### 2.6.2 Cell cycle block

By increasing retinoblastoma expression and decreasing cyclin A, cyclin B, and MDM2 expression, LA (5–50 μM) caused the cell cycle arrest of MKN-28, AGS, and MKN-45 cells at the G2/M transition (Xiao et al., 2011; Lin et al., 2017).

### 2.7 Colon cancer

Globally, colon cancer is the second most common cancer in women and the third most common cancer in men with slightly increased case numbers in men (O'Keefe, 2016). Licochalcones can induce apoptosis and inhibit proliferation in colon cancer cells (Keshavarz-Fathi and Rezaei, 2021).

#### 2.7.1 Induction of apoptosis

LA (10–40 μM) can enhance the production of intracellular ROS by inhibiting the expression of thioredoxin reductase-1, which activates the mitochondrial apoptosis pathway and induces apoptosis in HCT-116 cells (Wu et al., 2020).

#### 2.7.2 Cell cycle block

LA(IC_50_ = 7 and 8.8 µM, respectively) can prevent the hypoxia-induced proliferation of SW480 and SW620 cells by inhibiting the TrkB/AKT signaling pathway (Arita et al., 2020). LA (5–25 μmol/L) blocked the cell cycle of HCT116 cells at the G1 phase by increasing the level of p21 *via* inhibition of the JNK1 signaling pathway (Yao et al., 2014).

### 2.8 Bladder cancer

Bladder cancer is the 10th most common form of cancer worldwide (Bray et al., 2018). Research has shown that licochalcones can induce apoptosis of bladder cancer cells, inhibit their proliferation, and activate the immune system (Keshavarz-Fathi and Rezaei, 2021).

#### 2.8.1 Induction of apoptosis

By increasing intracellular Ca^2+^ and ROS levels, decreasing mitochondrial membrane potential, upregulating Apaf-1, caspase-9, caspase-3, and cleaved PARP levels, and elevating the Bax/Bcl-2 ratio, LA (10–80 μM) induced T24 cells apoptosis (Yang et al., 2016; Hong et al., 2019). LB (40–80 μM) induced apoptosis in T24 and EJ cells *via* decreases in Bcl-2 and survivin expression and enhanced expression of Bax, cleaved PARP, and caspase-3 (Yuan et al., 2014). LC (10–50 μM) induced apoptosis in T24 cells by decreasing Bcl-2, Bcl-w, and Bcl-xL expression levels while increasing Bax and Bim expression levels (Wang et al., 2015).

#### 2.8.2 Cell cycle block

By downregulating cyclin A, cyclin B1, and Wee1 expression and upregulating the expression of the cyclin-dependent kinase inhibitor p21WAF1/CIP1, LA (10–60 μM) blocked the cell cycle of T24 cells at the G2/M phase (Jiang et al., 2014; Hong et al., 2019). Via decreases in CDK1, CDK2, CDC25A, and CDC25B expression, LB (40–80 μM) led to cell cycle arrest of T24 and EJ cells at the S phase (Yuan et al., 2014).

#### 2.8.3 Activation of the immune system

In C3H/HeN mice carrying UM-UC-3 cells, LA (40 mg/kg) enhanced the activity of cytotoxic T lymphocytes and increased the number of CD4^+^ CD25^+^ Foxp3+ regulatory T cells. This suggests that LA can be used to treat bladder cancer by modulating the immune response (Zhang et al., 2016).

### 2.9 Cervical and ovarian cancer

In women, the incidence and mortality rates of cervical and ovarian cancers are lower than those of breast, colorectal, and lung cancers (Bray et al., 2018). Licochalcones can induce autophagy and apoptosis and inhibit the proliferation of cervical and ovarian cancer cells (Keshavarz-Fathi and Rezaei, 2021).

#### 2.9.1 Induction of autophagy

LA (10–50 μM) induced autophagy in SiHa and HeLa cervical cells by increasing the levels of LC3-II, Beclin1, ATG5, ATG7, and ATG12 through inhibition of the PI3K/Akt/mTOR signaling pathway (Tsai et al., 2015).

#### 2.9.2 Induction of apoptosis

By inhibiting the PI3K/Akt/mTOR signaling pathway, LA (10–50 μM) also induced apoptosis in SiHa, HeLa, CaSki, and C33A cervical cells *via* upregulation of caspase-3, caspase-9, and cleaved PARP levels and downregulation of Bcl-2 levels (Tsai et al., 2015). In addition, LA (25–50 μM) induces apoptosis in HeLa cells by increasing the expression of TRAIL-R2 (Szliszka et al., 2012). Via induction of ROS production, loss of mitochondrial transmembrane potential, cytochrome c release, increases in Bid and Bax levels, decreases in survivin, Bcl-2, and p53 levels, and activation of the caspase cascade, LA (5–25 μM) induced also the apoptosis in OVCAR-3 and SK-OV-3 ovarian cancer cells (Lee et al., 2012; Kim et al., 2013).

#### 2.9.2 Cell cycle block

LA (IC_50_ = 10.7 µM) prevented the hypoxia-induced proliferation of HeLa S3 cells by inhibiting the TrkB/AKT signaling pathway (Arita et al., 2020).

### 2.10 Glioma

Glioma is one of the most common primary malignancies of the adult central nervous system (Chen et al., 2021). Licochalcones can induce apoptosis and inhibit the proliferation, migration, and invasion of gliomas (Keshavarz-Fathi and Rezaei, 2021).

#### 2.10.1 Induction of apoptosis

By reducing the mitochondrial membrane potential and the production of ATP, LA (2–12.5 μM) induced mitochondrial fragmentation and caspase-3, -8, and -9 expression in glioma stem cells including GS-Y01, GS-Y03, U87GS, GS-NCC01, and A172GS cells (Kuramoto et al., 2017).

#### 2.10.2 Cell cycle block

By reducing the cyclin A, cyclin B1, cyclin E1, CDK1, CDK2, and CDK4 expression levels, LA (5–40 μM) induced the cell cycle arrest of glioma U87 cells at the G2/M phase (Lu et al., 2018).

#### 2.10.3 Inhibition of migration and invasion

By downregulating ADAM9 expression through inhibited activation of the MEK/ERK signaling pathway, LA (10–50 μM) prevented the migration and invasion of human gliomas including M059K, U-251 MG, and GBM8901 cells (Huang et al., 2018).

### 2.11 Sarcomas

Sarcomas are a rare, heterogeneous group of malignant tumors of the bone or soft tissue (Walczak and Irwin, 2013). Research has shown that licochalcones can induce autophagy and apoptosis and inhibit the proliferation, migration, and invasion of sarcomas (Keshavarz-Fathi and Rezaei, 2021).

#### 2.11.1 Induction of autophagy

LA (5–20 μmol/L) induced autophagy in A375 and B16 cells by increasing the expression of LC3-II, Beclin1, ATG5, and p62 *via* activation of the miR-142-3p/Rheb/mTOR signaling pathway (Zhang et al., 2020). By upregulating the LC3A/B-II level, LA (10–40 μM) induced autophagy in osteosarcoma HOS cells (Shen et al., 2019). By suppressing the PI3K/AKT/mTOR pathway, LB (5–20 μM) increased the expression of ARPs (ATG7, Beclin1) and promoted the p62 and LC3B decomposition turnover in MG-63 and U2OS cells, thereby inducing autophagy (Huang and Jin, 2022).

#### 2.11.2 Induction of apoptosis

By activating the miR-142-3p/Rheb/mTOR signaling pathway, LA (5–20 μmol/L) activated the caspase cascade and induced apoptosis in A375 and B16 cells (Chen et al., 2019; Zhang et al., 2020). In addition, by activating the extrinsic (i.e., upregulation of CHOP, DR4, and DR5 expression) and intrinsic (i.e., inhibition of Sp1 expression) apoptosis pathways, LA (5–20 μM) induced mitochondrial dysfunction, endoplasmic reticulum stress, and ultimately apoptosis in A375 melanoma cells (Kang et al., 2017b). Via increases in caspase-8, caspase-3, and cleaved PARP expression levels and decreases in Bcl-2, XIAP, and survivin expression levels, LA (10–40 μM) induced apoptosis in HOS cells (Shen et al., 2019). By activating the p38 MAPK signaling pathway, increasing the expression of Bax, cleaved PARP, caspase-3, -8, and -9, and decreasing the expression of Bcl-2, LA (20–100 μM) induced apoptosis in osteosarcoma 143B cells (Lin et al., 2019b). Via activation of the death receptor pathway (i.e., increased DR4 and DR5 expression), endoplasmic reticulum stress (i.e., increased CHOP expression), and mitochondrial apoptosis pathway (i.e., increased Bax and cleaved PARP expression and decreased Bcl-xL, Mcl-1, survivin, and Bcl-2 expression), LA (10–40 μM) induced apoptosis in malignant pleural mesothelioma MSTO-211H and H28 cells (Kim et al., 2015). Moreover, LD (20–80 μmol/L) induced the apoptosis in A375 cells by causing a loss in mitochondrial membrane potential, increasing ROS production and Bax, caspase-9, and caspase-3 expression, and downregulating the expression of Bcl-2 (Si et al., 2018).

#### 2.11.3 Cell cycle block

LA (10–40 μM) induced the cell cycle arrest of HOS cells at the G2/M transition by decreasing the levels of CDC2 and CDC25C *via* activation of the ATM/Chk2 checkpoint pathway (Shen et al., 2019). Moreover, LA (10–40 μM) blocked the cell cycle of MSTO-211H and H28 cells at the G1 phase by inhibiting cyclin D1 expression (Kim et al., 2015). LA (5–20 μM) also led to cell cycle arrest of HT-1080 cells at the G2 phase by decreasing the levels of CDK1 and CDK2 *via* inhibition of R132C-mutant isocitrate dehydrogenase 1 (Hu et al., 2020). Furthermore, LA (IC_50_ = 10.7 µM) inhibited hypoxia-induced proliferation of the neuroblastoma cell lines SK-N-SH, TGW, and GOTO by inhibiting the TrkB/AKT signaling pathway (Arita et al., 2020).

#### 2.11.4 Inhibition of migration and invasion

By decreasing the expression of MMP-2 and MMP-9, LD (20–80 μmol/L) inhibited the migration and invasion of A375 cells (Si et al., 2018).

## 3 Inhibition of the efflux of anticancer drugs

BCRP is an ATP-binding cassette transporter that has an important influence on the metabolism of anticancer drugs. Inhibition of BCRP expression can promote increased intestinal (re)uptake of antineoplastic drugs and decrease their hepatic metabolization, thereby enhancing their bioavailability. In addition to BCRP functions in normal tissues, the overexpression of this transporter in tumors causes multidrug resistance to anticancer drugs such as mitoxantrone, flavonol, and methotrexate (Steinbach et al., 2002; Volk et al., 2002; Tamaki et al., 2010). By reducing the expression of BCRP, LA (10–100 μM) reduced the BCRP-mediated efflux of doxorubicin and temozolomide in BCRP-MDCKII cells. Molecular docking analyses showed that Pi-Pi stacked interactions and potential Pi-Alkyl interactions play important roles (Fan et al., 2019). P-glycoprotein is also an important efflux protein, which mediates the drug resistance of a variety of cancer cells. LA (50–100 μM) reversed the resi stance of KB/MDR1 cells to vinblastine by inhibiting the activity of P-glycoprotein (Nabekura et al., 2015). By inhibiting c-Met overexpression, LA (Chen et al., 2014; Choi et al., 2014; Huang et al., 2014; Tang et al., 2016; Hirsch et al., 2017; Qiu et al., 2017; Chen et al., 2018; Niu et al., 2018; Wang et al., 2018; Lin et al., 2019a; Oh et al., 2019a; Wu et al., 2019; Gao et al., 2021; Heng and Cheah, 2021; Park et al., 2021; Yuan et al., 2021) decreased the activity of HCC827 and PC-9 cells and decreased gefitinib resistance in non-small cell lung cancer cells (Han et al., 2022). For H1975 gefitinib-resistant non-small cell lung cancer cells, LA (10–100 μM) reduced their drug resistance by decreasing Hsp90 activity through binding to the N-terminal ATP binding site of Hsp90 (Seo, 2013).

## 4 Review and speculation of the structure-activity relationship

Chalcones are widely recognized as privileged structures, families of molecules featuring scaffolds that upon appropriate decoration can lead to a large spectrum of diverse biological effects. Rossi et al. inserted substituents with different electronic and steric properties into ring B, and altered their positions on the aromatic core, resulting a new range of derivatives. These derivatives induced a remarkable block in G2/M phases, earlier and higher than LA (Rossi et al., 2022). Additionally, approximating to paclitaxel, a series of derivatives of LA (benzimidazole-2-substituted phenyl or pyridine-propylenone) showed significantly better anti-proliferative activity against HCT116, MCF-7 and HepG2 cells than 5-fluorouracil (Wu et al., 2016). Studies have shown that various synthetic LA derivatives are more cytotoxic to cancer cells than naturally occurring licochalcones. Bromo-retrochalcone and trifluoromethyl-retrochalcone can be used as antineoplastic drugs for pleural mesothelioma and oral squamous cell carcinoma with stronger anticancer activity than LA (Yoon et al., 2018). A derivative of LA (LA-linked thiazole-imidazopyridine) has greater antitumor activity than etoposide in MCF-7 and MDA MB-231 breast cancer cells, A549 lung cancer cells, and DU-145 prostate cancer cells (Suma et al., 2020). This indicates that structural modifications of licochalcones to increase their anticancer efficacy are promising.

## 5 Conclusion

**FIGURE 2:**
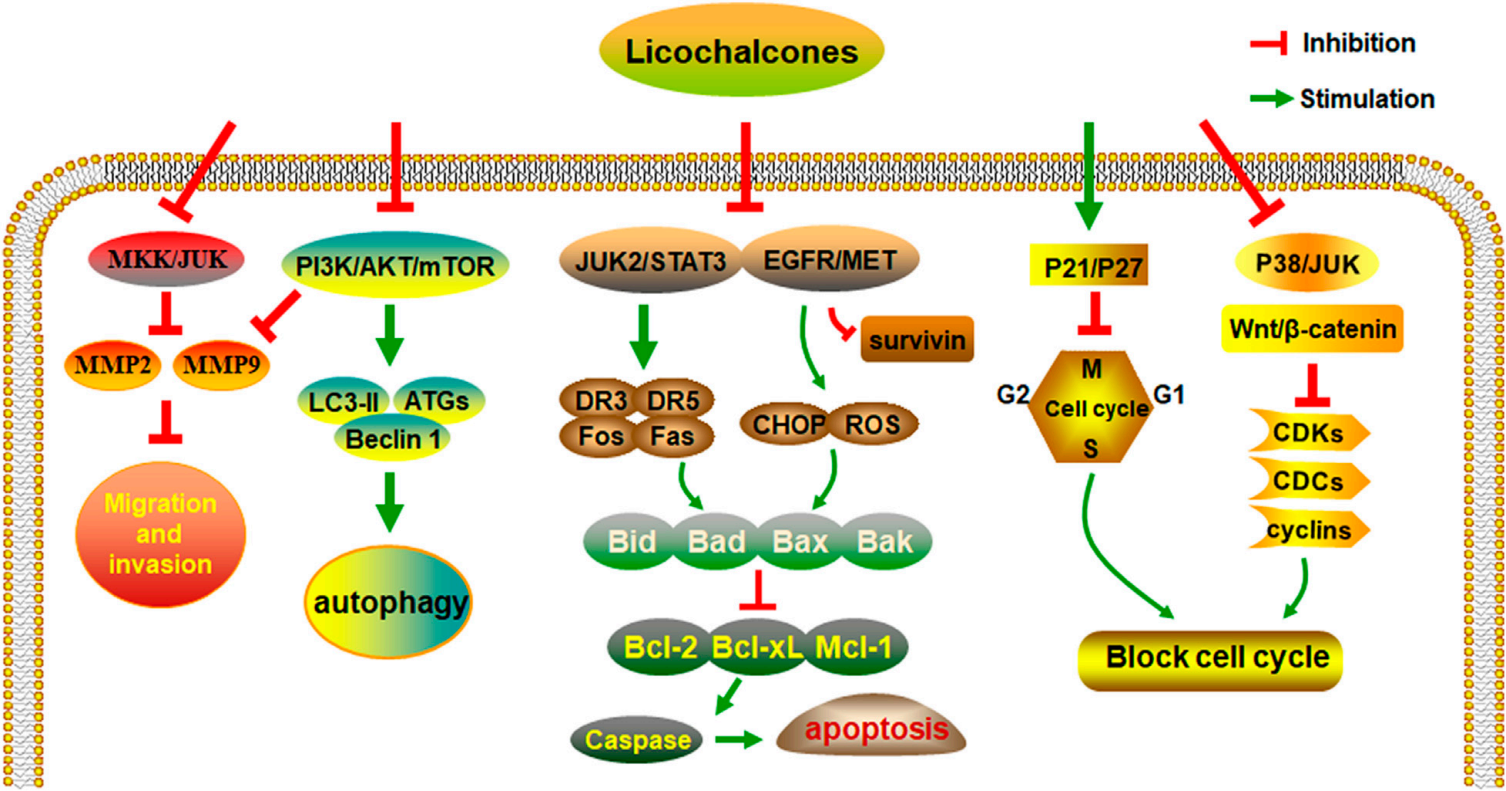
The anti-cancer activities and molecular mechanisms of licochalcones.

Studies have shown that licochalcones have a wide range of anticancer activities, such as in gastric, lung, colon, breast, liver, and bladder cancer. Among all licochalcones, LA has the most substantial antitumor effect. Although LA does not have such effects on all types of cancers, it still shows great potential for anticancer treatment. After analyzing and collating the literature, we conclude that the regulation of multiple signaling pathways by licochalcones including the EGFR/ERK, PI3K/Akt/mTOR, p38/JNK, JAK2/STAT3, MEK/ERK, Wnt/β-catenin, and MKK4/JNK signaling pathways is the key to their antineoplastic effects (Figure 2).

Among the examined licochalcones, LA not only has antineoplastic effects but also can be used to reduce drug efflux from cancer cells and reduce adverse reactions caused by other antitumor drugs (Herbrechter et al., 2015). Therefore, we believe that the use of LA as an adjunct to anticancer drugs holds great promise. Although licochalcones have demonstrated their value as antitumor drugs, most of these studies are in the cell experiment stage. More clinical studies are needed to confirm the antineoplastic effects of licochalcones. Moreover, except for LA, studies of other licochalcones regarding their anticancer potential are insufficient, making it necessary to further explore the antitumor mechanisms of other licochalcones.

## Data Availability

The original contributions presented in the study are included in the article/Supplementary Material, further inquiries can be directed to the corresponding author.
