# Supplementary material for: Anticancer effects of licochalcones: A review of the mechanisms
Source: Front Pharmacol. 2023 Jan 23;14:1074506. doi: 10.3389/fphar.2023.1074506 (PMC9900005; doi:10.3389/fphar.2023.1074506)
Supplement: Supplementary file 1 [file DataSheet1.docx]

Supplementary Material

Table 1. Abbreviation

| 4EBP1 | 4E-binding protein 1 |
| --- | --- |
| ADAM9 | A disintegrin and metalloprotease domain 9 |
| Apaf-1 | Apoptotic protease activating factor-1 |
| ATF4 | Activating transcription factor 4 |
| ATG | Autophagy-related gene |
| Atg13 | Autophagy-related 13 |
| ATM | Ataxia telangiectasia mutated serine/threonine kinase |
| ATP | Adenosine-triphosphate |
| Bad | Bcl-2-associated death promoter |
| Bak | Bcl-2 homologous killer |
| Bax | Bcl-2-associated X |
| Bcl-xL | B-cell lymphoma extra-large |
| BRCP | Breast cancer resistance protein |
| CDC | Cell division cycle gene |
| CDK | Cyclin-dependent kinases |
| CHEK2 | Checkpoint kinase 2 |
| CHOP | C/EBP homologous protein |
| c-Met | Tyrosine-protein kinase Met |
| CTMP | Chemithermomechanical pulp |
| DR3 | Death receptor 3 |
| DR5 | Death receptor 5 |
| EGFR | Epidermal growth factor receptor |
| eIF2 | Eukaryotic initiation factor 2 |
| eIF2α | Eukaryotic translation initiation factor 2α |
| ERK | Extracellular signal-regulated kinase |
| Fas | Factor associated suicide |
| Fos:Bid | BH3 interacting death agonist |
| GLUT1 | Glucose transporter 1 |
| Heat HSP90 | Heat shock protein 90 |
| IFN-γ | Interferon-γ |
| JAK2 | Janus kinase 2 |
| JNK | Jun N-terminal kinase |
| JUN | Jun proto-oncogene |
| LC3-II | Light chain 3‑II |
| MAPK | Mitogen-activated protein kinase |
| Mcl-1 | Myeloid cell leukemia-1 |
| MDM2 | Mouse doubleminute 2 homolog |
| MKK4 | Mitogen-activated protein kinase kinase 4 |
| MMPs | Matrix metalloproteinases |
| mTOR | mammalian target of rapamycin |
| NF-κB | Nuclear factor κB |
| PI3K | Phosphoinositide 3-kinase |
| p38 | p38 mitogen-activated protein kinase |
| p53 | tumor protein p53 |
| p70S6k | 70-kDA S6 protein kinase |
| PARP | Poly ADP-ribose polymerase |
| PDK1 | Phosphoinositide-dependent protein kinase 1 |
| PD-L1 | Programmed cell death-ligand 1 |
| PERK | PKR-like endoplasmic reticulum kinase |
| PKCε | Protein kinase C epsilon |
| PLCγ1 | Phospholipase Cγ1 |
| PP2A | Protein phosphatase 2A |
| PRAS40 | Proline-rich Akt substrate of 40kDa |
| PUMA | p53 upregulated modulator of apoptosis |
| ROS | Reactive oxygen species |
| Sp1 | Specificity protein 1 |
| STAT3 | Signal transducer and activator of transcription 3 |
| TNBC | Triple negative breast cancer |
| TNFR1 | Tumornecrosisfactorreceptor1 |
| TRAIL | Tumor necrosis-related apoptosis-inducing ligand |
| Trk-B | Tropomyosin-related kinase B |
| TSC1/2 | Tuberous sclerosis complex |
| ULK1 | Unc-51 like kinase 1 |
| uPA | Urokinase-like plasminogen activator |
| XIAP | X-linked inhibitor of apoptosis protein. |

Table 2. The anti-cancer effects of licochalcones

| Type of cancer | Ingredient | Detail | Cell lines | Dosage | References |
| --- | --- | --- | --- | --- | --- |
| Lung cancer | LA | Promoting LC3-II, ATG5, and ATG7 production | A549 and H1299 cells | 10-15 μM | [7] |
|  | LA | Increasing the LC3-II/LC3-I ratio and P62 level | A549 cells | 10-40 μM | [8] |
|  | LA | inhibiting hypoxia-induced HIF-1α accumulation and the expression of target genes GLUT1 and PDK1. | H1299 and H322 cells | 2.5-25 μM | [10] |
|  | LA | Decreasing the levels of Bcl-xL and Bcl-2, and increasing the levels of p-EIF2, ATF4 Bad, Bax, cleaved PARP, and caspase-3 | H460 and A549 cells | 10-15 μM | [11] |
|  | LA | Inhibiting the expression of MDM2, Cyclin B1, CDC2 and CDC25C | H460 and A549 cells | 10-15 μM | [11] |
|  | LA | Decreasing the levels of Bcl-2 and full-length PARP and increasing the level of cleaved PARP | A549 cells | 10-40 μM | [7, 8] |
|  | LA | Enhancing the expression of miR-144-3p and CHOP. | H292 cells | 10 μM | [9] |
|  | LA | Inhibiting the EGFR signaling pathway and the downstream kinases ERK1/2 and AKT | H3255, HCC827, H1975, and A549 cells | 5-50 μM | [12] |
|  | LA | Inhibiting Wnt/β-catenin signaling pathway | SK-LU-1 and NCI-H1703 cells | 10-50 μg/mL | [14] |
|  | LA | Reducing the levels of MMP-1 and MMP-3 by inhibiting AKT signaling pathway | A549 and H460 cells | 2-20 μM | [15] |
|  | LA | Inhibiting the phosphorylation of 4EBP1 and activation of PERK-eIF2α pathway by inhibiting the production of PD-L1 | A549 cells | 10-50 μM | [16] |
|  | LB | Inhibiting the EGFR and MET signaling pathway and induced mitochondrial dysfunction and endoplasmic reticulum stress | HCC827 cells | 5-15 μM | [13] |
|  | LB | Decreasing the cyclinB1 and CDC2 protein expression, and increasing p27 expression | HCC827 cells | 5-15 μM | [13] |
| Liver cancer | LA | Activating the mitochondrial apoptosis pathway and promoting the expression of Bax, Bad, and caspase-3 | HepG2 cells | 5-20 μM | [17] |
|  | LA | Inducing ROS accumulation and the expression of CHOP, caspase-4, -9, and -3 | HepG2 cells | 1-50 μM | [18] |
|  | LA | Increasing the expression of DR3, DR5, Fas and Bad, Bax, Bak, PUMA, and caspases-3, and decreasing the expression of survival factor PKCε, p70S6K | HepG2 cells | 30-70 μM | [19] |
|  | LA | Increasing the expression of Weel, P21, and JNK1 and decreasing the expression of survivin, cyclin B1, Cyclin D1, and CDK1 | HepG2 cells | 30-70 μM | [19, 25] |
|  | LA | Activating the ULK1/Atg13 signaling pathway and then increasing the expression of TSC1/2, PRAS40, CTMP, PP2A | HepG2 cells | 5-50 μM | [20] |
|  | LA | Inhibiting uPA and MMP9 expression through inhibiting the MKK4/JNK and NF-κB signaling pathways | HA22T/VGH and SK-Hep-1 cells | 5-20 μM | [27, 28] |
|  | LA | regulating MAPK signaling pathway and FoxO signaling pathway | HepG2 cells | 70 μM | [21] |
|  | LB | Increasing the expression of TNF-R1, Fas, Fas-L, caspases-8, JUN, Fos, Bak, caspase-9, and caspases-3 | HepG2 cells | 10-120 μM | [22] |
|  | LB | Decreasing the expression of CDK1, Cyclin B1, CHK2, CDC14B, and CDC7 and increasing the expression of p21 | HepG2 cells | 10-120 μM | [22] |
|  | LB | regulating miR-29b-3p and miR-96-5p miRNAs | HepG2 cells | 120 μM | [23] |
|  | LB | increased p27 expression and decreased the levels of cyclin B1 and Cdc2 | HepG2 and Huh7 cells | 10–20 μM | [26] |
|  | LD | Increasing the expression of Bid, Bad and cleaved PARP, and reducing the expression of Bcl-xl and Mcl-1 | HCC827 cells | 5-20 μM | [24] |
|  | LD | Decreasing the levels of cyclin B1 and CDC2 and increasing the expression of p21 and p27, | HCC827 cells | 5-20 μM | [24] |
| Breast cancer | LA | Increasing the expression of LC3‑II by inhibiting the PI3K/Akt/mTOR signaling pathway | MCF-7 cells | 5-50 μM | [29] |
|  | LA | Reducing the expression of Bcl-2, promoting the expression of Bax and caspase-3 by inhibiting the PI3K/Akt/mTOR signaling pathway | MCF-7 cells | 5-50 μM | [30] |
|  | LA | Reducing cyclin D1 expression and increasing the p21 expression | MCF-7 cells | 5-50 μM | [30] |
|  | LA | Promoting the expression of p21, p27, Bid, Bcl-xl, and cleaved PARP, and decreasing the expression of Bcl-2, Mcl-1 and survivin by inhibiting the expression of Sp1 | MCF-7 and MDA-MB-231 cells | 5-50 μM | [31] |
|  | LA | Increasing the level of acylcarnitine and inhibiting the expression of prostaglandin reductase 1 | HCC38 TNBC cells | 1-100 μM | [32] |
|  | LA | Reducing the expression of cyclin D1 | MCF-7 cells | 5-15 μg/ml | [34] |
|  | LA | Inhibiting the expression of PRMT6 and then p53 expression | MCF-7 cells | 10-100 μM | [36] |
|  | LA | Inhibiting the expression of E-cadherin and vimentin | MDA-MB 231 cells | 5-40 μΜ | [30] |
|  | LB | Increasing the expression of Bid, Bad, cleaved PARP and caspase-3, and decreasing the expression of Bcl-2 | MCF-7 cells | 10-50 μM | [33] |
|  | LB | Decreasing the expression of cyclin A, CDK2, and CDC25A, and increasing the level of p21 | MCF-7 cells | 10-50 μM | [33] |
|  | LE | Decreasing the expression of VEGF-A, uPA, MMP-9, CDK4, CDK2, cyclin A, and cyclin D1 | MDA-MB 231 cells xenograft mice | 7-14 mg/kg | [37] |
| Oral carcinoma | LA | Increasing the expression of p27, p21, Bax, Bid, Bcl-xl, cleaved PARP, and caspase-3 and decreasing the cyclin D1 | HN22 and HSC4 cells | 10-40 μM | [39] |
|  | LA | Activating the FasL-mediated death receptor pathway and increasing the levels of caspase-8 and -3 | KB cells | IC_50_=50 μM | [41] |
|  | LA | Activating the death receptor pathway, and increasing the expression of p53, Bad, Bax, caspase-3, -8, and -9, and release of cytochrome c | FaDu cells | 10-100 μM | [42, 43] |
|  | LA | Reducing the levels of IGF-1, MMP-2, and MMP-9 by inhibiting the PI3K/AKT signaling pathway | 25-100 μM | SCC4 and CAL-27 cells | [50] |
|  | LA | Reducing MMP-2 expression, and increasing the levels of TIMP and E-cadherin | SCC-25 | 25-100 μg/mL | [51] |
|  | LB | Increasing the expression of DR 4, DR5, CHOP, Bax, cleaved PARP, and caspase-3, and decreasing the expression of survivin, Bcl-xl, and Mcl-1 | HN22 and HSC4 cells | 10-30 μM | [44] |
|  | LC and LD | Increasing the expression of DR4, DR5, ROS, CHOP, p21, Bax, Bid, Apaf-1 and cleaved PARP and down-regulating the expression of Bcl-2, Mcl-1, and survivin by inhibiting the JAK2/STAT3 signaling pathway | HN22 and HSC4 cells | 10-50 and 10-30 μM, respectively | [45, 46] |
|  | LB and LD | Increasing the levels of p21 and p27, and decreasing the level of cyclin D1 | HN22 and HSC4 cells | 10-30 μM | [44, 46] |
|  | LD | Increasing the expression of Fas-L, p53, Bax, Bid, and caspase-3, and decreasing the expression of Bcl-2 | FaDu cells | 12.5-50 μg/ml | [47] |
|  | LH | Down-regulating the expression of Bcl‑2 and Bcl-xL and up-regulating the expression of Bax and Bad by inhibiting the Matr3 | HSC2 and HSC3 cells | 10-30 μM | [48] |
|  | LH | Activating death receptor pathway, endoplasmic reticulum stress, and mitochondrial apoptosis pathway by inhibiting the JAK/STAT3 signaling pathway | HN22 and HSC4 cells | 5-20 μM | [49] |
|  | LH | Increasing the levels of p21 and p27, and decreasing the level of cyclin D1 by inhibiting JAK/STAT3 signaling pathway | HN22 and HSC4 cells | 5-20 μM | [49] |
| Esophageal cancer | LB | Inhibiting the JAK2/STAT3 signaling pathway and decreasing the target protein Mcl-1 expression | KYSE450 and KYSE510 cells | 5-20 μM | [53] |
|  | LC | Inducing endoplasmic reticulum stress and activating mitochondrial apoptosis pathway by inhibiting the JNK and p38 MAPK signaling pathway | KYSE 30, 70, 410, 450, and 510 cells | 10-50 μM | [54] |
|  | LH | Increasing the expression of DR4, DR5 ROS, CHOP, p21, Bax, Bid, Apaf-1, and cleaved PARP and decreasing the expression of Bcl-2, Mcl-1, and survivin | KYSE 30 and KYSE 450 cells | 5-20 μM | [55] |
|  | LH | Down-regulating the expression of CDC2, cyclin B1 and increasing the expression of p21 and p27 | KYSE 30 and KYSE 450 cells | 5-20 μM | [55] |
| Gastric cancer | LA | Promoting the intracellular ROS generation and activating the mitochondrial apoptosis pathway and caspase cascade | BGC-823 cells | 20-100 μM | [56] |
|  | LA | Increasing the expression of Bax, Bad, cleaved PARP, caspases-3, -8, and -9, and reducing the expression of Bcl-2, cyclin A, cyclin B, and MDM2 | GES-1, MKN-28, SGC7901, AGS and MKN-45 cells | IC_50_=92.7, 42.0, 40.8, 41.1 and 40.7 μM, respectively. | [57, 58] |
|  | LA | Down-regulating the expression of hexokinase 2A and inhibiting the glycolysis | MKN45 and SGC7901 cells | 10-50 μM | [59] |
| Colon cancer | LA | Enhancing the production of ROS by inhibiting the expression of thioredoxin reductase-1 in | HCT-116 cells | 10-40 μM | [61] |
|  | LA | Inhibiting the TrkB-AKT signaling pathway | SW480 and SW620 cells | IC_50_=7 and 8.8 µM, respectively | [62] |
|  | LA | Increasing the level of p21 by inhibiting the JNK1 signaling pathway. | HCT116 cells | 5-25μmol/L | [63] |
| Bladder cancer | LA | Increasing the levels of Apaf-1, caspase-9 and caspase-3, PARP cleavage and Bax/Bcl-2 ratio | T24 cells | 10-80 μM | [64, 65]. |
|  | LB | Decreasing the expression of CDK1, CDK2, Cdc25A, and Cdc25B, Bcl-2, and survivin, and enhancing the expression of Bax, cleaved PARP and caspase-3 | T24 and EJ cells | 40-80 μM | [66] |
|  | LC | Decreasing the expression of Bcl-2，Bcl-w and Bcl-xL and increasing the expression of Bax and Bim | T24 cells | 10-50 μM | [67] |
|  | LA | Inhibiting the expression of Cyclin A, Cyclin B1, Wee1 and increasing p21WAF1/CIP1 expression | T24 cells | 10-60 μM | [65, 68] |
|  | LA | Enhancing the activity of cytotoxic T lymphocyte and count of CD4+ CD25+ Foxp3+T cells | UM-UC-3 cells xenograft mice | 40 mg/kg | [69] |
| Glioma | LA | Reducing the mitochondrial membrane potential and the production of ATP, and induced mitochondrial fragmentation and the expression of caspase-3, -8 and -9 | GS-Y01, GS-Y03, U87GS, GS-NCC01, and A172GS | 2-12.5 μM | [75] |
|  | LA | Reducing the expression of cyclin A, cyclin B1, cyclin E1, CDK1, CDK2, and CDK4 | U87 cells | 5-40 μM | [76] |
|  | LA | Inhibiting the ADAM9 expression through inhibiting the activation of the MEK-ERK signaling pathway | M059K, U-251 MG and GBM8901 cells | 10-50 μM | [77] |
| Sarcoma | LA | Increasing the expression of LC3-II, beclin1, ATG5, and p62 by activating the miR-142-3p/Rheb/mTOR signaling pathway | A375 and B16 cells | 5-20 μmol/L | [79] |
|  | LA | Increasing the levels of LC3A/B-II, caspase-3, and cleaved PARP, and decreasing expression of Bcl-2, XIAP, survivin, CDC2, and CDC25C | HOS cells | 10-40 μM | [80] |
|  | LA | Up-regulating the expression of CHOP, DR4, and DR5 and inhibiting the expression of Sp1 | A375 cells | 5-20 μM | [83] |
|  | LA | Increasing the expression of Bax, cleaved PARP, caspase-3, -8, and -9, and decreasing the expression of Bcl-2 | 143B cells | 20-100 μM | [84] |
|  | LA | Increasing the expression of CHOP, DR4, DR5, Bax, and cleaved PARP, and decreasing the expression of cyclin D1, Bcl-xL, Mcl-1, survivin, and Bcl-2 | MSTO-211H and H28 cells | 10-40 μM | [85] |
|  | LA | Decreasing the levels of CDK1 and CDK2 by inhibiting the activity of R132C-mutant isocitrate dehydrogenase 1. | HT-1080 cells | 5-20 μM | [87] |
|  | LB | elevated the expression of ARPs (ATG7, Beclin1) and promoted the p62 and LC3B decomposition turnover | MG-63 and U2OS cells | 5-20 μM | [81] |
|  | LD | Increasing the expression of Bax, caspase-9, and caspase-3, and down-regulating the expression of Bcl-2, MMP-2, and MMP-9 | A375 cells | 20-80 μmol/L | [86] |
